# Analysis of circRNAs and circRNA-associated competing endogenous RNA networks in β-thalassemia

**DOI:** 10.1038/s41598-022-12002-0

**Published:** 2022-05-16

**Authors:** Fang Yang, Heyun Ruan, Shuquan Li, Wei Hou, Yuling Qiu, Lingjie Deng, Sha Su, Ping Chen, Lihong Pang, Ketong Lai

**Affiliations:** 1 Department of Obstetrics and Gynecology, The First Affiliated Hospital of Guangxi Medical University, Nanning, Guangxi China; 2 Department of Obstetrics and Gynecology, Minzu Hospital of Guangxi, Zhuang Autonomous Region, Affiliated Minzu Hospital of Guangxi Medical University, Nanning, Guangxi China; 3 NHC Key Laboratory of Thalassemia Medicine, The First Affiliated Hospital of Guangxi Medical University, Nanning, Guangxi China; 4 Key Laboratory of Thalassemia Medicine, Chinese Academy of Medical Sciences, The First Affiliated Hospital of Guangxi Medical University, Nanning, Guangxi China; 5 Guangxi Key Laboratory of Thalassemia Research, The First Affiliated Hospital of Guangxi Medical University, Nanning, Guangxi China

**Keywords:** Anaemia, Disease genetics

## Abstract

The involvement of circRNAs in β-thalassemia and their actions on fetal hemoglobin (HbF) is unclear. Here, the circRNAs in β-thalassemia carriers with high HbF levels were comprehensively analyzed and compared with those of healthy individuals. Differential expression of 2183 circRNAs was observed and their correlations with hematological parameters were investigated. Down-regulated hsa-circRNA-100466 had a strong negative correlation with HbF and HbA_2_. Bioinformatics was employed to construct a hsa-circRNA-100466‑associated competing endogenous RNA (ceRNA) network to identify hub genes and associated miRNAs. The hsa-circRNA-100466▁miR-19b-3p▁SOX6 pathway was identified using both present and previously published data. The ceRNA network was verified by qRT-PCR analysis of β-thalassemia samples, RNA immunoprecipitation of K562 cell lysates, and dual-luciferase reporter analysis. qRT-PCR confirmed that hsa-circRNA-100466 and SOX6 were significantly down-regulated, while miR-19b-3p was up-regulated. Hsa-circRNA-100466, miR-19b-3p, and SOX6 were co-immunoprecipitated by anti-argonaute antibodies, indicating involvement with HbF induction. A further dual-luciferase reporter assay verified that miR-19b-3p interacted directly with hsa-circRNA-100466 and SOX6. Furthermore, spearman correlation coefficients revealed their significant correlations with HbF. In conclusion, a novel hsa-circRNA-100466▁miR-19b-3p▁SOX6 pathway was identified, providing insight into HbF induction and suggesting targets β-thalassemia treatment.

## Introduction

β-Thalassemia is a monogenetic disorder, most prevalent in specific geographical areas, including the Mediterranean region, parts of Africa, and Southeast Asia^1^. It is caused by the defect in the β-globin gene on chromosome 11, leading to reduced hemoglobin levels and anemia, with the severity determined by the extent of impaired β-globin synthesis^2^. Heterozygous individuals with one defective allele may have no symptoms or only mild symptoms, while double heterozygosity or homozygosity results in β-thalassemia major (β-TM) or β-thalassemia intermedia (β-TI). Patients with β-TI usually have moderate anemia that does not require transfusion support, while β-TM patients are severely anemic and require lifelong transfusion and iron chelation therapy to survive^3^. An effective treatment is the use of hematopoietic stem cells, however, suitable related and matched donors are often difficult to find^4,5^ and are lacking for most patients^6^.

Fetal hemoglobin (HbF), containing two α-globin and two γ-globin chains, predominates in the fetus and newborn. Shortly after birth, β-globin is expressed in place of γ-globin. In the normal adult, HbF comprises < 1.0% of the total hemoglobin. However, in pathological states such as β-thalassemia, the expression of HbF may increase. HbF is recognized as a critical modulator of clinical symptoms in β-thalassemia patients^7^. Elevated synthesis of the γ-globin chain effectively alters the imbalance of the α-/β- globin chains, alleviating the clinical symptoms^8^. Therapies targeting HbF induction may thus be a promising approach for treating β-thalassemia.

Circular RNAs (circRNAs) are non-coding RNAs that may be encoded by either exons or introns^9^. CircRNAs have been implicated in a variety of processes, including hematopoiesis and hematological diseases^10,11^. What is more, circRNA-based diagnostic and therapeutic strategies show great potential. However, there is scant information on the role of circRNAs in diseases related to hemoglobin, and nothing is known of their functions in β-thalassemia.

In this study, we isolated nucleated red blood cells (NRBCs) and reticulocytes from the peripheral blood of β-thalassemia carriers with high HbF as well as healthy controls. We then analyzed the circRNAs in these cells, their expression profiles, and their potential functions in HbF induction. It is hoped that these results may provide a novel reference for further research on the regulatory function of circRNAs on HbF in β-thalassemia.

## Results

### Clinical characteristics of participants

The participants were assigned to two groups according to their hemoglobin and hematological characteristics. The high-HbF-β-thalassemia carrier group (F) included 20 participants (HbF > 5.0%, HbA_2_ > 3.5%) while the control group (C) included 20 healthy individuals. The mean HbF and HbA_2_ values are listed in Table 1, which differed significantly between the F and C groups. The F and C groups had similar age and sex distributions.

**Table 1:** Characteristics of the subjects.

Items	The high-HbF group	The control group	P value
Number	N = 20	N = 20	
**Sex**			
Female	11	9	> 0.05
Male	9	11	> 0.05
Age (mean ± SD)	33.90 ± 7.26	32.65 ± 6.88	> 0.05
HbF (mean ± SD)	9.66 ± 3.65	0.60 ± 0.19	< 0.001
HbA_2_ (mean ± SD)	4.86 ± 0.79	2.71 ± 0.22	< 0.001

### CircRNA profiles determined by microarray

**Figure 1:**
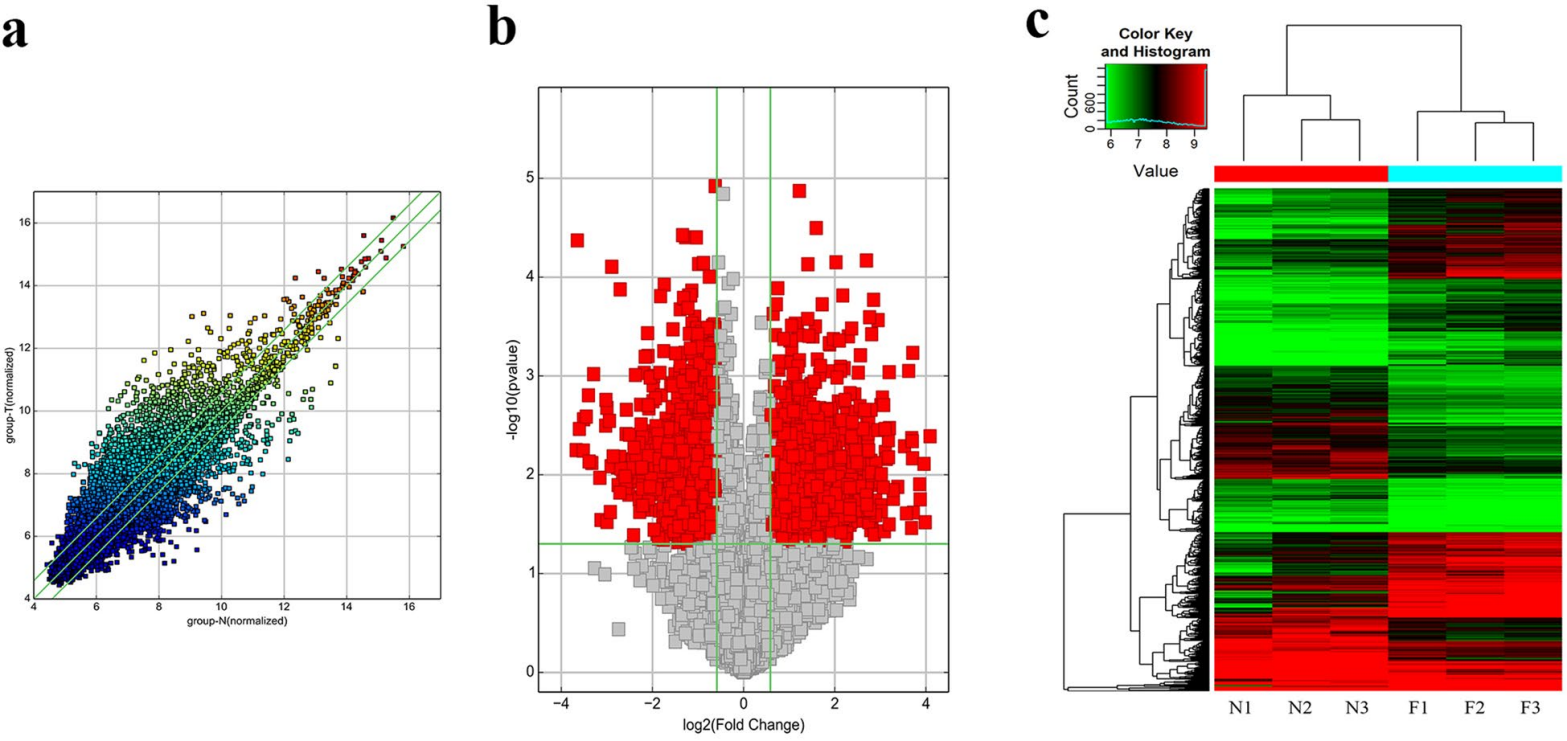
The expression profiles of circRNAs compared between β-thalassemia carriers with high HbF and healthy controls. (**a**) Scatter plot showing the distribution of circRNA expression. (**b**) Volcano plots showing differential expression of circRNAs. (**c**) Clustergram showing the entire circRNA expression profiling of the samples.

To identify the circRNAs associated with HbF in β-thalassemia, we investigated circRNAs in three pairs of healthy subjects and high-HbF-β-thalassemia carriers by circRNA microarray. ArrayStar Human circRNA Array analysis was adopted for profiling the human circRNAs expression. The fold changes (FC) in circRNA expression were determined, showing the presence of 2183 circRNAs with fold changes ≥ 1.5 and significantly different expression (P < 0.05) between the F and C groups. Of these, 1209 circRNAs were up-regulated and 974 were down-regulated (Fig. 1a–c). The circRNA expression profile data are available in the GEO databases (accession number GSE196682). GO and KEGG analyses were performed based on the KEGG database^12,13^ (Supplementary Figure S1).

### Identification of HbF-related circRNAs in β-thalassemia

**Figure 2:**
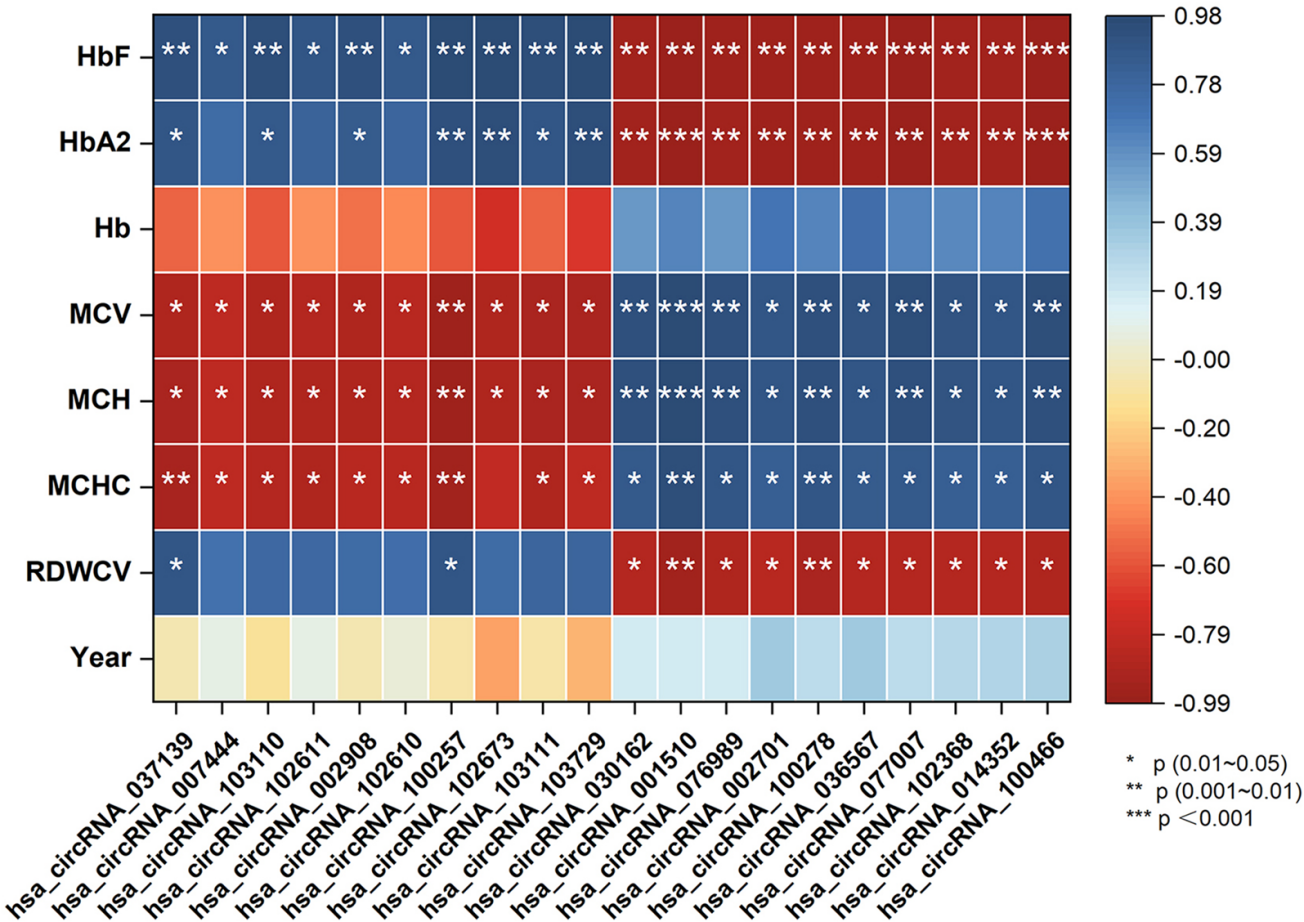
Heatmap showing the correlation between circRNAs expression and hematologic parameters. The top 10 essential nodes ranked by MCC scores were determined by CytoHubba plugin.

Pearson’s correlation analysis was used to evaluate the association between circRNA expression and hematological parameters. Positive circRNAs were defined as those whose expression showed significant association with hematological parameters, showing significant association HbF, HbA_2_, Hb, MCV, MCH, MCHC and RDWCV (|Pearson R| > 0.8 and P < 0.05, Supplementary Dataset). A circRNA whose expression value correlated with HbF (|Pearson R| > 0.9 and P < 0.01) was defined as a significant HbF-related circRNA. The top 10 positive HbF-correlated and negative HbF-correlated circRNAs are shown in the heatmap (Fig. 2). The down-regulated circRNA, hsa-circRNA-100466, showed a strong negative correlation with both HbF and HbA_2_ (P < 0.001) and was thus analyzed further.

### Construction of the circRNA-associated ceRNA network

To explore the role of hsa-circRNA-100466 in β-thalassemia, we established a ceRNA regulatory network centered on hsa-circRNA-100466. This was based on the premise that circRNAs sponge miRNAs to modulate mRNA activity.

**Figure 3:**
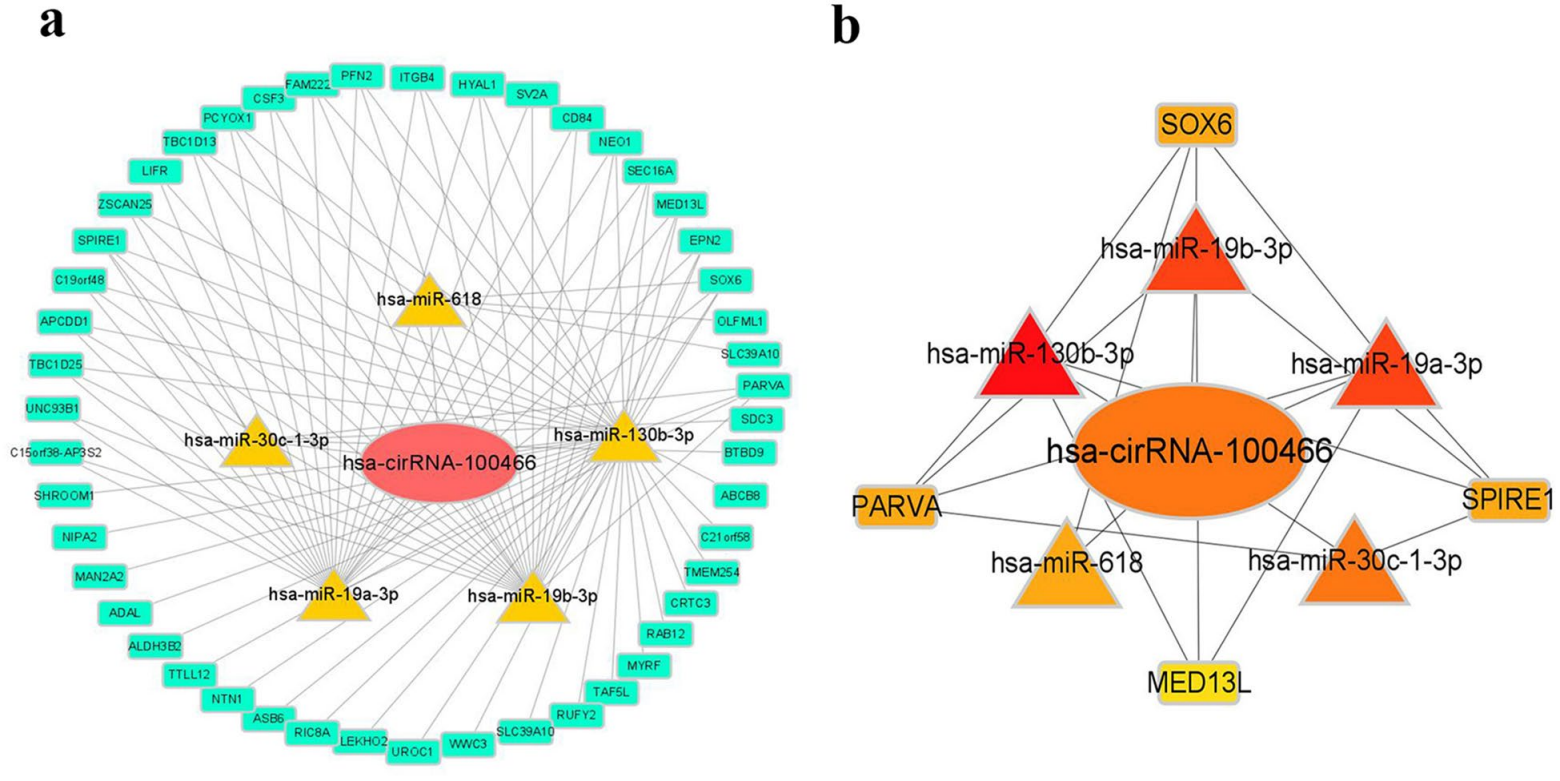
Hsa-circRNA-100466 serves as a sponge for multiple miRNAs. (**a**) Network of circRNA/miRNA/mRNA interactions based on hsa-circRNA-100466. Red ellipse node, circRNA; yellow triangle node, up-regulated differentially expressed miRNA; green rectangle type node, mRNA. (**b**) A sub-ceRNA network based on hub RNAs.

The ceRNA network identified 7 miRNA nodes and 28 mRNA nodes (Fig. 3a). We further analyzed the hub genes and miRNAs using the “Cytohubba” plugin. This led to the final identification of five hub miRNAs (miR-19b-3p, miR-19a-3p, miR-130b-3p, miR-618, and miR-30c-1-3p). Four hub genes, SOX6, PARVA, SPIRE1, and MED13L, were also identified. As shown in the sub-ceRNA network, hsa-circRNA-100466 modulated the expression of the hub genes through binding to (miR-19b-3p, miR-19a-3p, miR-130b-3p, miR-618, and miR-30c-1-3p (Fig. 3b). These predicted interactions offer insight into HbF induction.

### Verification of the ceRNA network

Firstly, we used dataset GSE93973 from our previous microarray studies in which aberrantly expressed miRNAs were identified in β-thalassemia patients with high HbF compared to healthy controls. The five hub miRNAs observed in the present study were also detected in GSE93973, but only miR-19b-3p met the differential expression criteria of P < 0.05 and was identified as a differentially expressed miRNA. Meanwhile, the hub gene SOX6 encodes a transcription factor that plays an important role in erythropoiesis. Therefore, the hsa-circRNA-100466▁miR-19b-3p▁SOX6 pathway was selected for further verification.

**Figure 4:**
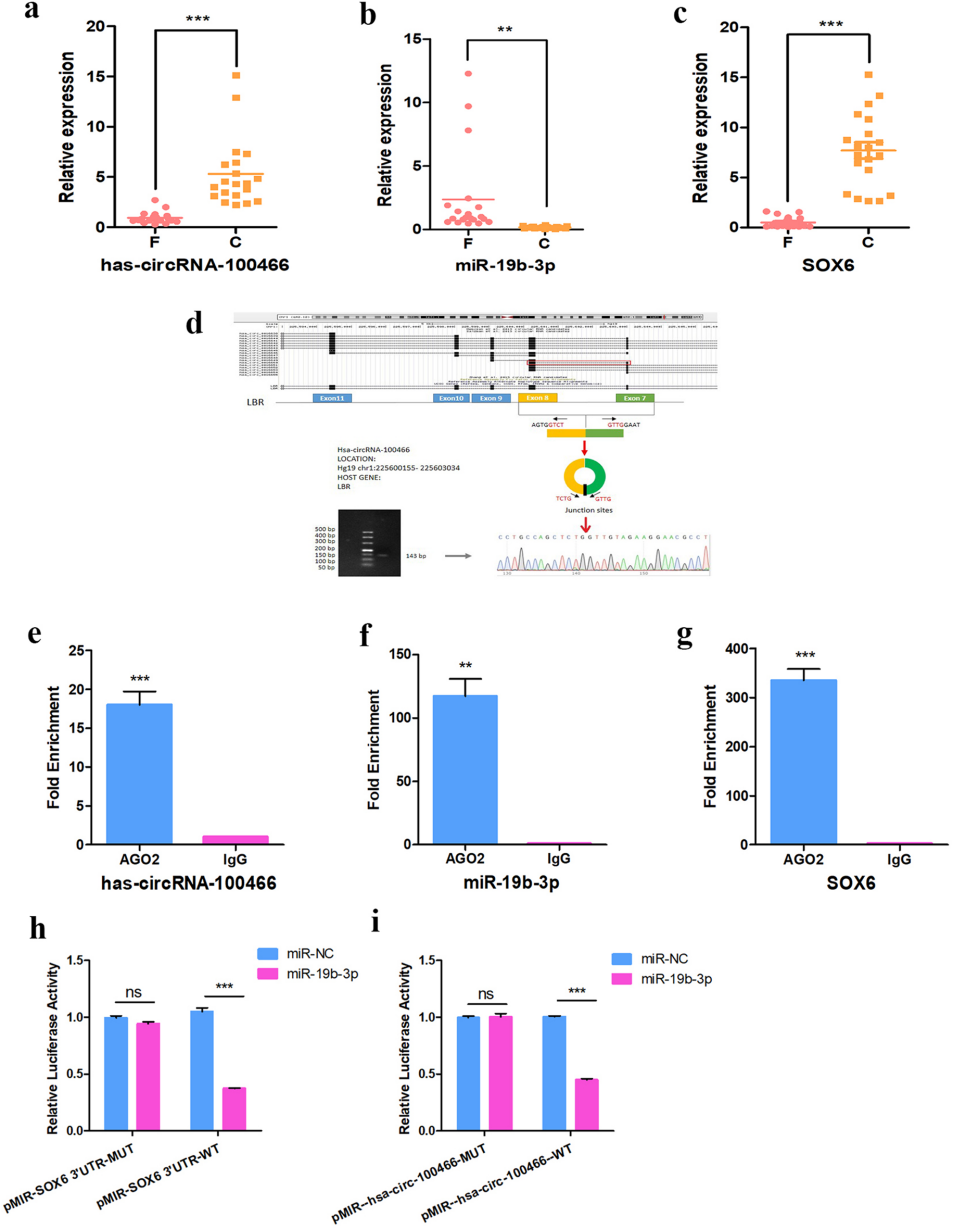
Verification of the ceRNA network by qRT-PCR, RIP, and dual-luciferase reporter assay. The expression levels of hsa-circRNA-100466 (**a**) and SOX6 (**c**) were significantly down-regulated in the F group in comparison with the C group, whereas miR-19b-3p expression levels (**b**) were significantly up-regulated. Schematic diagram showing the location of hsa-circRNA-100466 on chromosome 1:225,600,155–225,603,034 and its derivation from LBR exons 7–8. Agarose gel electrophoresis and Sanger sequencing results of hsa-circRNA-100466 qRT-PCR products (**d**). RIP assay of hsa-circRNA-100466 in K562 cells. Hsa-circRNA-100466 (**e**), miR-19b-3p (**f**), and SOX6 (**g**) were significantly pulled down by the anti-AGO2 antibody. Dual-luciferase reporter assay showing that miR-19b-3p mimics significantly repressed the luciferase activity of hsa-circRNA-100466-WT but not the mutant (**h**). Dual-luciferase reporter assay showing that miR-19b-3p mimics significantly repressed the luciferase activity of SOX6 3’-UTR-WT but not the mutant (**i**). **P < 0.01; ***P < 0.001.

Next, qRT-PCR was used to verify the differential expression of hsa-circRNA-100466, miR-19b-3p, and SOX6 in the F and C groups. The results were consistent with the microarray data, showing down-regulation of hsa-circRNA-100466 and SOX6 and up-regulation of miR-19b-3p in the F group (Fig. 4a–c). The size of the hsa-circRNA-100466 qRT-PCR product was verified by agarose gel electrophoresis. Sanger sequencing of the qRT-PCR products confirmed the back-splicing junction sites in hsa-circRNA-100466, which is consistent with the circBase database (Fig. 4d).

Numerous investigations of the interactions between miRNAs and mRNAs have suggested a pivotal role for Argonaute2 (AGO2), which has been shown to form complexes with miRNAs and thereby modulate their actions^14^. To investigate this, RIP on K562 cell extracts was performed using an anti-Ago2 antibody. In the RIP experiment, hsa-circRNA-100466 was found to significantly enriched by the AGO2 antibody compared with the IgG control (Fig. 4e), suggesting that hsa-circRNA-100466 is a potential ceRNA. It was also found that both miR-19b-3p and SOX6 mRNA were specifically associated with AGO2, with no enrichment seen with the control IgG (Fig. 4f, g), indicating that miR-19b-3p may sponge SOX6.

A subsequent dual-luciferase reporter assay revealed that the luciferase intensity was markedly reduced after the co-transfection of the pMIR-hsa-circRNA-100466-WT luciferase reporter and miR-19b-3p mimics, whereas the mutated luciferase reporter did not have such effect (Fig. 4h, i). The dual-luciferase reporter assay also showed that the miR-19b-3p mimics significantly reduced the luciferase activity of SOX6 3’-UTR-WT but not the mutant in 293T cells. This suggested that hsa-circRNA-100466 bound to miR-19b-3p, and that SOX6 was targeted by miR-19b-3p.

### Association between hsa-circRNA-100466/miR-19b-3p/SOX6 and HbF levels

**Figure 5:**
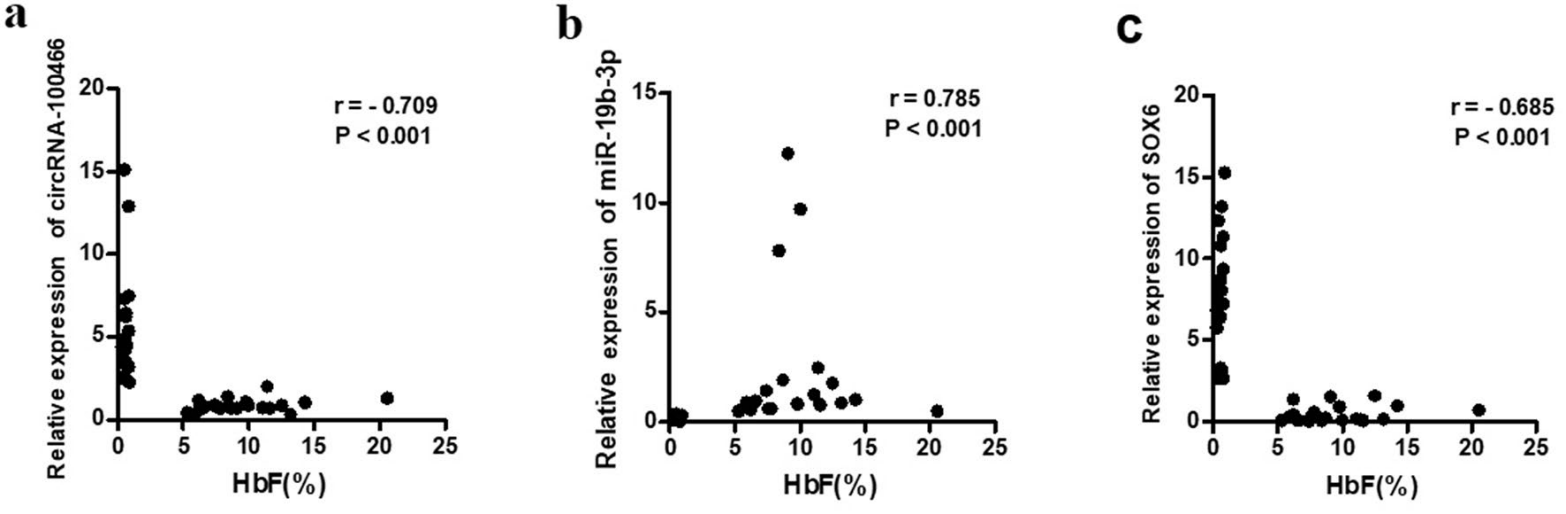
Associations between hsa-circRNA-100466/miR-19b-3p/ SOX6 and HbF levels. The Spearman correlation coefficient was utilized to evaluate associations between (**a**) hsa-circRNA-100466 and HbF, (**b**) miR-19b-3p and HbF, and (**c**) SOX6 and HbF.

Spearman correlations were used to examine the relationships between the qRT-PCR-determined levels of hsa-circRNA-100466, miR-19b-3p, and SOX6 and HbF. While significantly positive correlations were seen between miR-19b-3p and HbF (r = 0.785, P < 0.001), significantly negative correlations were seen between hsa-circRNA-100466 and HbF (r = -0.709, P < 0.001), and SOX6 and HbF (r = -0.685, P < 0.001) (Fig. 5a–c).

## Discussion

Previous investigations of HbF induction in β-thalassemia have addressed transcriptional modulation and possible therapeutic targets such as miRNAs and long noncoding RNAs (lncRNAs). The roles of circRNAs have only recently been recognized, especially in relation to hematological disease. CircRNA-MYBL2 has been found to influence the progression of FLT3-ITD AML through modulation of FLT3 expression and has been suggested as a potential target for the treatment of the disease^15^. However, the roles of circRNAs in β-thalassemia are unknown. Here, we used microarray hybridization to identify differentially expressed circRNAs in patients with high HbF levels and to determine the roles of these circRNAs in HbF induction.

To the best of our knowledge, the present study is the first microarray investigation of circRNAs in NRBCs and reticulocytes from β-thalassemia carriers with high HbF levels compared with healthy controls. The study identified a total of 2183 abnormally expressed circRNAs. Further bioinformatics analysis was done to explore the relationship between these circRNAs and the hematological phenotype, by measurement of HbF, HbA_2_, Hb, MCV, MCH, MCHC and RDWCV. The bioinformatics predictions revealed a set of circRNAs that were significantly associated with the hematological phenotype. Of these, hsa-circRNA-100466 showed a 9.78-fold down-regulation in the F group and was thus strongly correlated with the HbF level. These results were verified by qRT-PCR, and were found to be consistent with the microarray findings. This suggests that hsa-circRNA-100466 may regulate the expression of genes responsible for HbF induction.

The circRNA field is relatively new, and there are no reports on the function of hsa-circRNA-100466. The evidence indicates that circRNAs function as ceRNAs, or sponges, binding miRNAs to regulate the expression of downstream target genes. For instance, circRNA-DLEU2 has been observed to play a vital role in AML tumorigenesis. Mechanistically, circRNA-DLEU2 was found to act as a sponge to adsorb miR-496, leading to elevated PRKACB expression^16^. CircRNA_0079480 was reported to sponge miR-654-3p, thereby promoting the progression of AML by increasing HDGF expression^17^.

Here, we identified hsa-circRNA-100466 as potentially associated with HbF induction and used it as the center of a ceRNA network. This identified five miRNA nodes and 28 mRNA nodes. The “CytoHubba” plugin in Cytoscape was then used to determine the important genes in the network^18,19^. The plugin offers 11 node ranking methods, such as MCC, and scores and ranks the nodes. These methods all showed that miR-19b-3p, miR-19a-3p, miR-130b-3p, and SOX6 were hub nodes, suggesting their central roles. MCC is generally considered to be better than the other methods. MCC identified five hub miRNAs and four hub mRNAs. Notably, the expression of miR-19b-3p was found to be significantly raised in our previous study (dataset GSE93973), where it was found to regulate HbF levels. Interestingly, the role of SOX6 in β-thalassemia has been documented and it is considered to be a repressor of γ-globin. SOX6 is a member of the Sox (Sry-type HMG box) transcription factor gene family; many of these proteins regulate cell-fate specification in many cell types, including erythroid cells^20,21^. SOX6 interacts directly with BCL11A, resulting in recruitment to the γ-globin gene regions, and has been found to prevent γ-globin transcription in erythroid progenitor cells^22^. Down-regulation of SOX6 was found to induce γ-globin expression in K562 cells and primary erythroid cells from β-thalassemia major patients and normal controls^23^. Increased γ-globin gene expression induced by Acyclovir (ACV) was also found to be linked to SOX6 down-regulation^24^. Collectively, these reports indicate the accuracy of our findings and suggest the importance of the has-circRNA-100466▁miR-19b-3p▁SOX6 pathway in HbF induction in β-thalassemia.

To further verify the sub-ce-network, qRT-PCR analysis was conducted in a larger sample. This demonstrated that miR-19b-3p levels were markedly increased in clinical β-thalassemia samples, while has-circRNA-100466 and SOX6 were correspondingly reduced, confirming the ceRNA hypothesis. To detect the ability of hsa-circRNA-100466 to sponge miR-19b-3p, RIP in K562 cell lysates was used. It is known that RNA-induced silencing complexes (RISCs) are formed by miRNA ribonucleoprotein complexes (miRNPs) in complex with AGO2^25,26^. Thus, immunoprecipitation of AGO2 will also identify miRNAs and their RNA interaction partners. Our RIP experiments confirmed that hsa-circRNA-100466 recognized and bound to AGO2. This suggests that hsa-circRNA-100466 exerts its regulatory functions through the classical method of binding to miRNAs^27^. Besides hsa-circRNA-100466, miR-19b-3p and SOX6 were also observed in the anti-AGO2 immunoprecipitates, verifying the interaction between hsa-circRNA-100466, miR-19b-3p, and SOX6 in HbF induction. A subsequent dual-luciferase reporter assay verified miR-19b-3p interacted directly with hsa-circRNA-100466 and SOX6. Together with the observed correlations between hsa-circRNA-100466, miR-19b-3p, and SOX6 expression and HbF levels, our findings strongly suggest that hsa-circRNA-100466 sponges miR-19b-3p to suppress SOX6 transcription, leading to increased HbF levels.

In summary, we have demonstrated the existence of a variety of differentially expressed circRNAs in β-thalassemia which may be involved in the induction of HbF and disease pathology. Furthermore, this is the first report of the role of hsa-circRNA-100466 in suppressing the transcription of SOX6 by sponging miR-19b-3p, thereby increasing HbF levels. These findings indicate that modulation of the hsa-circRNA-100466▁miR-19b-3p▁SOX6 pathway be a potential treatment for β-thalassemia.

These findings require confirmation by functional studies and future large-scale clinical trials. We intend to explore these aspects in future studies. It is hoped that these findings will offer a theoretical basis for determining the mechanism of β-thalassemia pathogenesis and may provide a direction for the development of treatment for β-thalassemia patients.

## Materials and methods

### Study participants

This study was conducted in accordance with the Declaration of Helsinki and was approved by the medical ethics committee of the First Affiliated Hospital of Guangxi Medical University. The participants were recruited from the First Affiliated Hospital of Guangxi Medical University. All participants provided written informed consent before commencement of the study. Hemoglobin electrophoresis, high-performance liquid chromatography (HPLC), and molecular analyses were used for α-thalassemia and β-thalassemia diagnosis. In all, 20 β-thalassemia carriers with high HbF levels (> 5.0%, HbA_2_ > 3.5%) and 20 age- and sex-matched healthy subjects with normal levels (< 2.0%) were enrolled. Subjects younger than 18 years of age, pregnant women, α-globin multiplications, non-deletional HPFH, histories of transfusions, or hematological malignancies were not recruited.

### RNA extraction from NRBCs and reticulocytes

**Figure 6:**
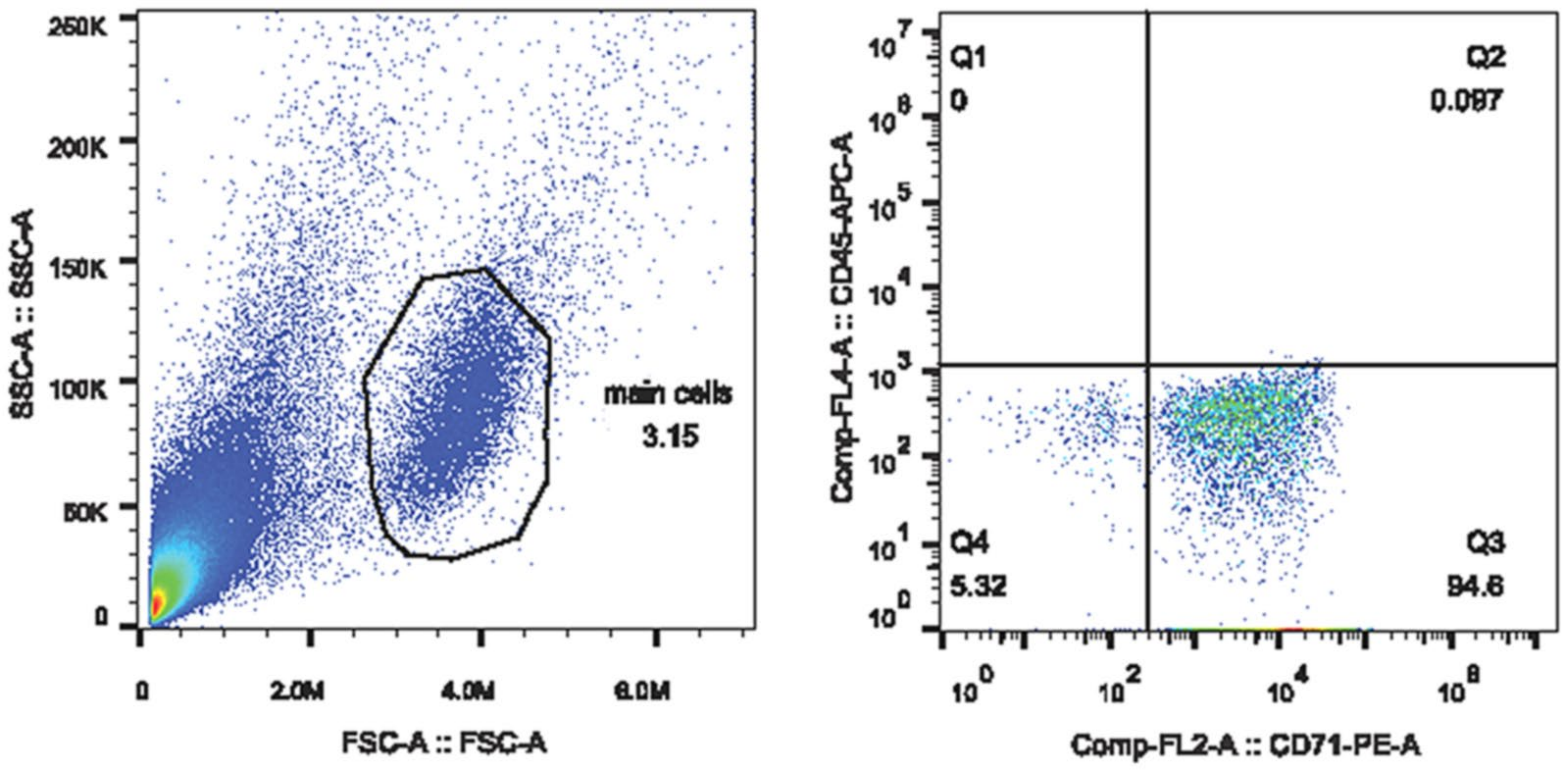
Confirmation of red blood cell sorting by fow cytometry. Aggregation of signals in the Q3 region is indicative of a mostly (94.6%) CD71^+^ cell population.

Ten milliliters of peripheral blood were collected into EDTA tubes and centrifuged at 500*g* for 2 min. Peripheral blood mononuclear cells were isolated from whole blood by density gradient centrifugation mononuclear cell separation media (Solarbio, Beijing, China). A negative CD45 (CD45 Microbeads human, Miltenyi Biotec GmbH, Germany) selection method with a magnetic shelf (MiniMACS Starting Kit, Miltenyi Biotec GmbH, Germany) was used to isolate NRBCs and reticulocytes^28^. The purity of isolated cells was determined using flow cytometry. The results of flow cytometry are showed in Fig. 6. RNA was extracted using TRIzol and RNAiso Plus (Takara, Shiga, Japan), per supplied protocol. The concentration and quality of the RNA were determined by spectrophotometry, using a NanoDrop ND-1000, and denaturing agarose gel electrophoresis, respectively.

### CircRNA microarray analysis

An Arraystar human circRNA array V2, containing 13 617 probes, was used for circRNA analysis, using the provided instructions for labeling and hybridization. Specifically, after amplification and transcription to fluorescent cRNA (Arraystar Super RNA Labeling Kit, Arraystar, Rockville, MD, USA), the cRNAs were hybridized on to the array and incubated at 65 °C in a hybridization oven (Agilent Technologies, Santa Clara, CA, USA) for 17 h. After washing and fixation, the arrays were scanned (Agilent DNA Microarray Scanner, part number G2505C) and imaged with Agilent Feature Extraction software (version 11.0.1.1). Analysis, including quantile normalization, was done with the “limma” package in R. CircRNAs with fold changes ≥ 1.5 and P values < 0.05 were considered differentially expressed.

### Correlations between circRNA expression and hematological parameters

Correlations between circRNA levels and the hematological parameters HbF, HbA_2_, Hb, MCV, MCH, MCHC and RDWCV were determined using Pearson’s correlation analysis. Absolute values |Pearson R| > 0.90 and P value < 0.05 were used for further analysis.

### Construction of a circRNA‑associated ceRNA network

Probable interactions between circRNAs and miRNAs were examined using Arraystar's home-made miRNA target prediction software based on TargetScan^29^ and miRanda^30^. The starBase v2.0^31^ (http://starbase.sysu.edu.cn/) and mirDIP^32^ (http://ophid.utoronto.ca/mirDIP/) databases were used for the prediction of mRNAs targeted by miRNAs. The ceRNA network was constructed and visualized by matching circRNA–miRNA and miRNA–mRNA pairs with Cytoscape v3.8.2. The “Maximal Clique Centrality” (MCC), a node ranking method offered by the “CytoHubba” plugin in Cytoscape was used for network analysis to identify hub genes and miRNAs.

### Verification of the differentially expressed circRNA and its related miRNA

qRT-PCR was used to verify the differentially expressed hsa-circRNA-100466, miR-19b-3p, and SOX6. cDNA was obtained from total RNA with Prime Script TMRT reagent Kit with GDNA Eraser (Takara Bio, Shiga, Japan). Next, quantitative RT-PCR (RT-qPCR) was performed using a QuantiNova SYBR Green PCR kit (QIAGEN, Germany), following the provided instructions. The β-actin and U6 were used for normalization of gene and miRNA expression, respectively. All primers (Supplementary Table S1) were designed and synthesized by Tsingke Biotechnology (Shanghai, China). Relative gene expression was determined using the comparative 2^−△△Ct^ methods. The size of the hsa-circRNA-100466 qRT-PCR product was verified by agarose gel electrophoresis, and the sequences of the back-splicing junction sites were verified by Sanger sequencing.

### Cell culture

K562 and 293T cell lines (Procell Biotechnology Co., Ltd, Wuhan, China) were grown in RPMI 1640 (Gibco, USA) with 10% fetal bovine serum (FBS) (Siji-qing, Beijing,China), and 1% penicillin–streptomycin (Solarbio, Zhejiang,China) in a humidified incubator at 37 ℃ with 5% CO_2_. Cells in logarithmic growth with 95% viability were used for experiments.

### RNA immunoprecipitation (RIP) assay

The Magna RIP RNA-Binding Protein RIP kit (Millipore, Bedford, MA, USA) was used in accordance with the supplied instructions. Briefly, lysates of 2 × 10^7^/l cells were incubated with magnetic beads conjugated with normal mouse IgG (control) or human anti-AGO2 (Millipore, Bedford, MA, USA). Briefly, lysates of 2 × 10^7^cells per liter were incubated with magnetic beads conjugated with 5 µg normal rabbit IgG (cat. #PP64B, Millipore, USA) or 5 µg rabbit anti-AGO2 (cat. #T56652, Abmar, China). The immunoprecipitated RNAs were isolated and verified using qRT-PCR.

### Dual-luciferase reporter assay

The wild/mutant sequence of hsa-circRNA-100466 or 3′-UTR of the SOX6 mRNA was cloned into the pMIR-REPORT luciferase vector and the pMIR-hsa-circRNA-100466-WT/MUT and pMIR-SOX6 3′-UTR-WT/MUT vectors were constructed (OBiO Technology, Shanghai, China). The cells (293T) were seeded and transfected with the pMIR-hsa-circRNA-100466-WT/MUT or pMIR-SOX6 3′-UTR-WT/MUT and miR-19b-3p mimics or miR-19b-3p mimics negative control following the instructions provided with the Lipofectamine 2000 reagent (Invitrogen, Waltham, MA, USA). The cells were incubated for 48 h, lysed, and the luciferase activity of each group was measured using the dual-luciferase reporter assay system (Promega, Madison, WI, USA), according to the supplied protocol. Data representing firefly luciferase activity were normalized against Renilla luciferase activity.

### Statistical analysis

All statistical data were analyzed with SPSS 20.0 software (IBM Corp., Armonk, NY, USA). Data are shown as means ± SD. Statistical analysis was performed using unpaired t-tests. All statistical tests were two-sided. P values < 0.05 were considered significant.

## Supplementary Information

Supplementary Information 1.

Supplementary Information 2.
