# Supplementary material for: Analysis of circRNAs and circRNA-associated competing endogenous RNA networks in β-thalassemia
Source: Sci Rep. 2022 May 16;12:8071. doi: 10.1038/s41598-022-12002-0 (PMC9110710; doi:10.1038/s41598-022-12002-0)
Supplement: Supplementary file 1 — Supplementary Information 1. [file 41598_2022_12002_MOESM1_ESM.docx]

**SUPPLEMENTARY DATA**

Figure S1. GO and KEGG pathway analysis of differentially expressed circRNAs.


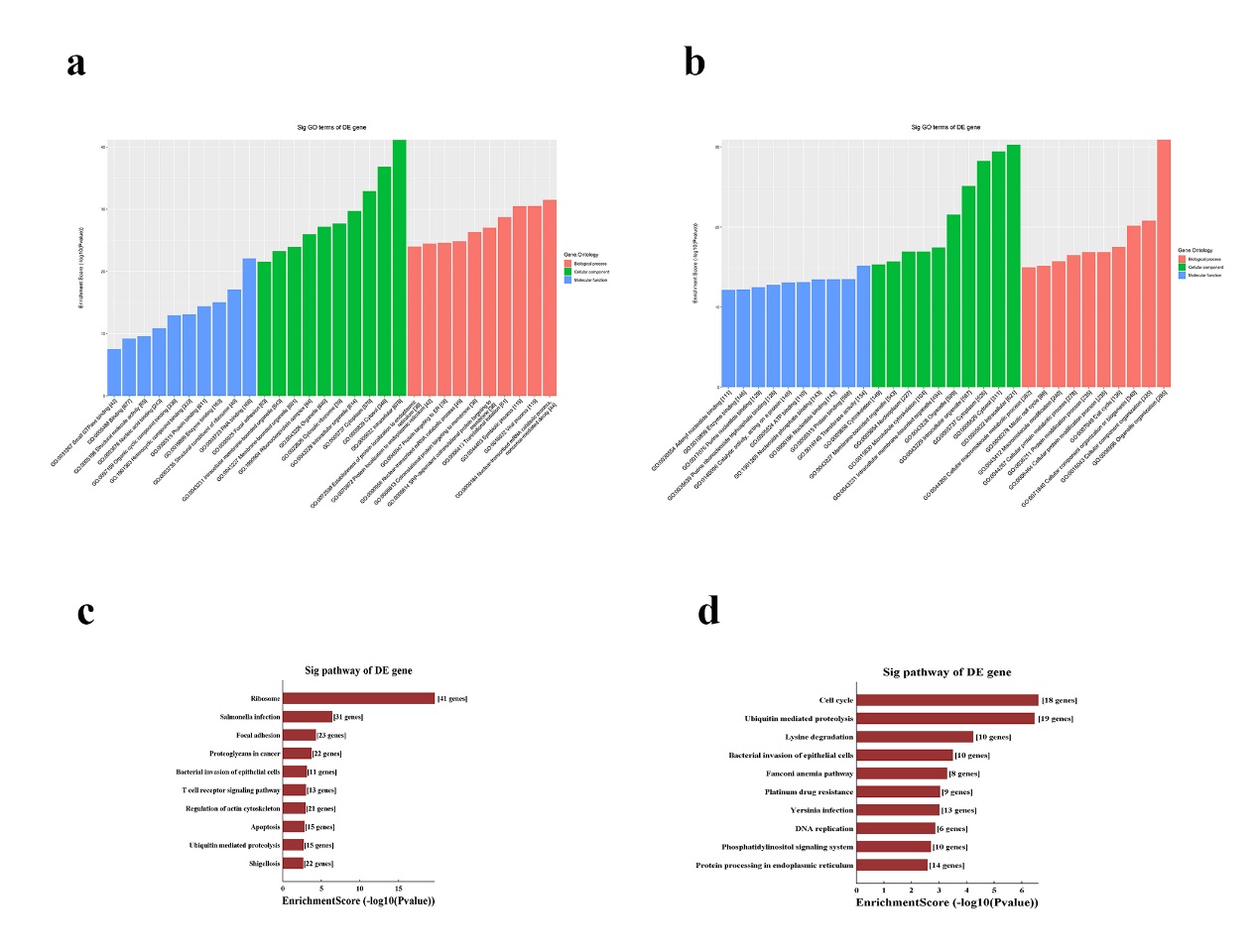


GO annotation of (A) down-regulated and (B) up-regulated circRNAs, via the top 10 enrichment scores-covering domains of biological processes, cellular components and molecular functions. The 10 significant pathways of (C) down-regulated and (D) up-regulated circRNAs by enrichment scores.

Figure S2. The Agarose gel electrophoresis result of the hsa-circRNA-100466 qRT-PCR product.


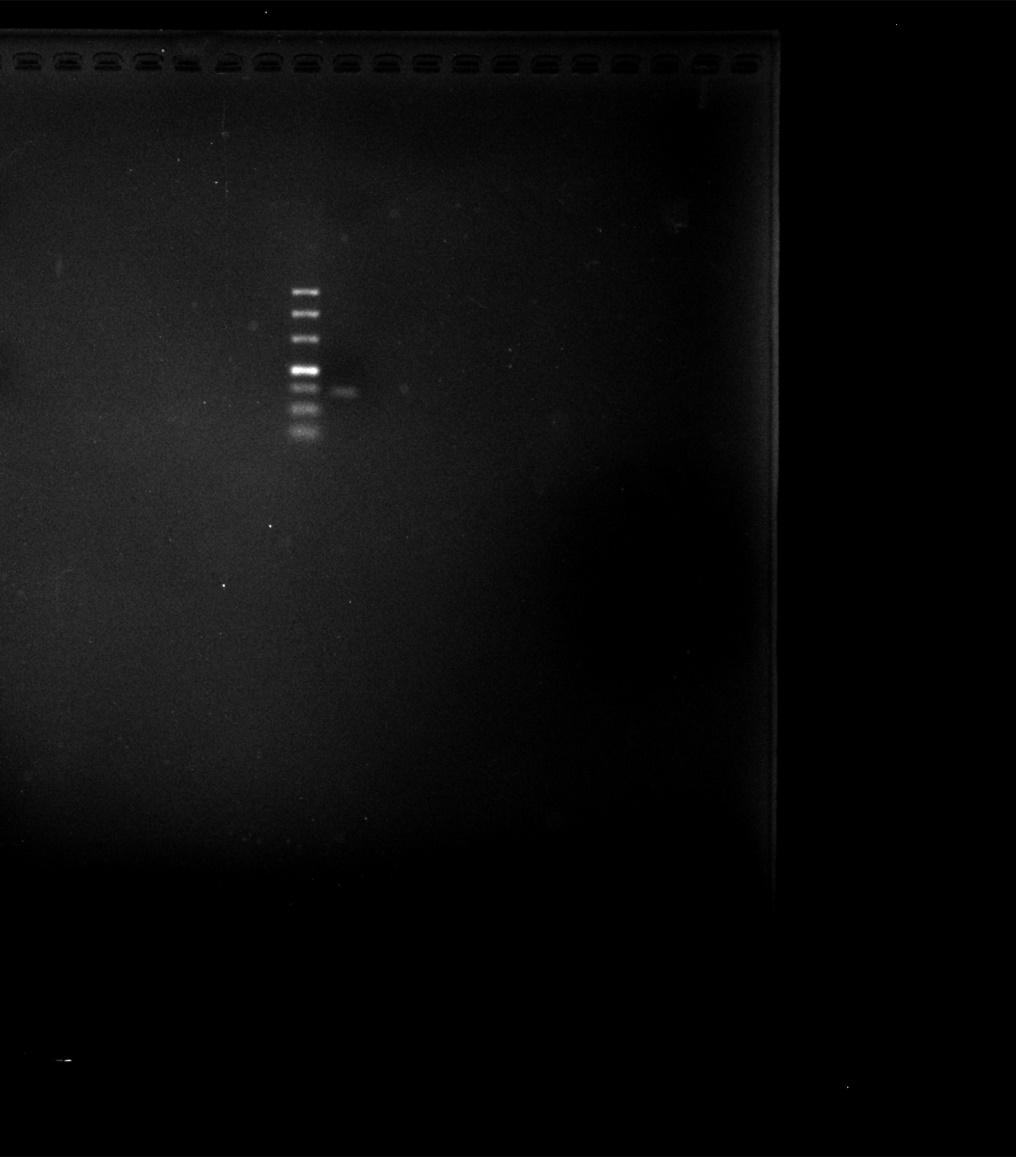


Supplementary Table S1: The sequences of the qRT-PCR primers

| Primer name | Sequence (5′-3′) | | Product length (bp) |
| --- | --- | --- | --- |
| β-actin（H） | forward | GTGGCCGAGGACTTTGATTG | 73 |
|  | reverse | CCTGTAACAACGCATCTCATATT |  |
| hsa_circRNA_100466 | forward | CACGTAATGAAACTCTACGCCC | 143 |
|  | reverse | GCCTGCCAGCTCTGGTTGT |  |
| SOX6 | forward | TACCTCTACCTCACCACATAAGC | 221 |
|  | reverse | ACATCGGCAAGACTCCCTTTG |  |
| hsa-miR-19b-3p | forward | CCGGCATTGTGCAAATCCATG | 65 |
|  | reverse | GTGCGTGTCGTGGAGTCG |  |
| U6 | forward | GCTTCGGCAGCACATATACTAAAAT | 89 |
|  | reverse | CGCTTCACGAATTTGCGTGTCAT |  |
